# Wzb of *Vibrio vulnificus* represents a new group of low-molecular-weight protein tyrosine phosphatases with a unique insertion in the W-loop

**DOI:** 10.1016/j.jbc.2021.100280

**Published:** 2021-01-12

**Authors:** Xin Wang, Qingjun Ma

**Affiliations:** 1 Key Laboratory of Experimental Marine Biology, Institute of Oceanology, Chinese Academy of Sciences, Qingdao, China; 2 Laboratory for Marine Biology and Biotechnology, Pilot National Laboratory for Marine Science and Technology, Qingdao, China; 3 University of Chinese Academy of Sciences, Beijing, China; 4 Center for Ocean Mega-Science, Chinese Academy of Sciences, Qingdao, China

**Keywords:** *Vibrio vulnificus*, low-molecular-weight protein tyrosine phosphatase (LMWPTP), tyrosine phosphorylation, Wzb, capsular polysaccharide, virulence factor, indel, protein structure, enzyme mechanism, infectious disease, phosphotyrosine, CPS, capsular polysaccharide, IPTG, isopropyl-β-D-thiogalactopyranoside, LMWPTP, low-molecular-weight protein tyrosine phosphatase, PEG, polyethylene glycol, pNPP, para-nitrophenyl phosphate, rmsd, root-mean-square deviation, TEV, tobacco etch virus

## Abstract

Protein tyrosine phosphorylation regulates the production of capsular polysaccharide, an essential virulence factor of the deadly pathogen *Vibrio vulnificus*. The process requires the protein tyrosine kinase Wzc and its cognate phosphatase Wzb, both of which are largely uncharacterized. Herein, we report the structures of Wzb of *V. vulnificus* (*Vv*Wzb) in free and ligand-bound forms. *Vv*Wzb belongs to the low-molecular-weight protein tyrosine phosphatase (LMWPTP) family. Interestingly, it contains an extra four-residue insertion in the W-loop, distinct from all known LMWPTPs. The W-loop of *Vv*Wzb protrudes from the protein body in the free structure, but undergoes significant conformational changes to fold toward the active site upon ligand binding. Deleting the four-residue insertion from the W-loop severely impaired the enzymatic activity of *Vv*Wzb, indicating its importance for optimal catalysis. However, mutating individual residues or even substituting the whole insertion with four alanine residues only modestly decreased the enzymatic activity, suggesting that the contribution of the insertion to catalysis is not determined by the sequence specificity. Furthermore, inserting the four residues into *Escherichia coli* Wzb at the corresponding position enhanced its activity as well, indicating that the four-residue insertion in the W-loop can act as a general activity enhancing element for other LMWPTPs. The novel W-loop type and phylogenetic analysis suggested that *Vv*Wzb and its homologs should be classified into a new group of LMWPTPs. Our study sheds new insight into the catalytic mechanism and structural diversity of the LMWPTP family and promotes the understanding of the protein tyrosine phosphorylation system in prokaryotes.

Phosphorylation on protein tyrosine is a posttranslational modification with essential roles in life. In eukaryotes, it has been extensively studied as an essential regulatory mechanism in various cellular processes (1). In contrast, protein tyrosine phosphorylation in prokaryotes was recognized much later than in eukaryotes (2). It regulates a couple of processes in prokaryotes, including polysaccharide synthesis, biofilm formation, and cell growth (3, 4, 5, 6). With technique developments in recent years, more proteins with various functions have been identified to be tyrosine-phosphorylated in bacteria and archaea (7, 8). Now it has been recognized that protein tyrosine phosphorylation is a key regulation paradigm in the physiology and pathology of prokaryotes (9, 10, 11). Yet, our knowledge on prokaryotic protein tyrosine phosphorylation system is very limited.

*Vibrio vulnificus* is the most deadly foodborne pathogen, causing gastroenteritis, wound infection, and severe septicaemia with an over 50% mortality rate (12). It is also an important pathogen for marine animals, causing huge economic loss in aquaculture industry (13). In *V. vulnificus*, protein tyrosine phosphorylation plays a key role in the production of capsular polysaccharide (CPS), one essential virulence factor for the pathogen to escape host immune response (14). The CPS synthesis gene locus of *V. vulnificus* is similar to that of the group 1 or 4 capsules of *Escherichia coli* (15). In *E. coli*, the relationship between tyrosine phosphorylation and CPS production has been basically elucidated. Three conserved proteins Wza-Wzb-Wzc are responsible for polymerization control and translocation of CPS (16). Specifically, Wza is the translocon of CPS, while Wzc and Wzb form a kinase/phosphatase pair required for high-level polymerization of polysaccharide (Fig. 1). Wzc belongs to the family of bacterial tyrosine kinases (BY kinases), a distinct class of kinases that have no homologs in eukaryotes. Wzc acts as an autokinase to phosphorylate its own tyrosine-rich C-terminal tail and other sites, resulting in a high phosphorylated Wzc, while the cognate phosphatase Wzb dephosphorylates Wzc to a low phosphorylation state. The cycling of Wzc between high and low phosphorylation states regulates the production of CPS, with the mechanism remaining elusive (16, 17). The exact situation in *V. vulnificus* is unknown yet but supposed to work in an analogous manner. Nevertheless, there are experiment evidences to directly connect tyrosine phosphorylation to CPS production in *V. vulnificus*. In studies of colony morphologies, Wzb of *V. vulnificus* (*Vv*Wzb) was found to be a switch of morphological transition among opaque, translucent, and intermediate phases through controlling CPS production (18). Deletion of *Vv*Wzb led to CPS production defect and attenuated virulence of the bacterium. It was also observed that the expression levels of Wzb and Wzc were correlated to CPS production (19). So far, *V. vulnificus* Wzb and Wzc have not been biochemically characterized yet, which hinders further understanding of the phosphorylation events in this deadly pathogen. In this study, we would focus on the characterization of *Vv*Wzb.

**Figure 1:**
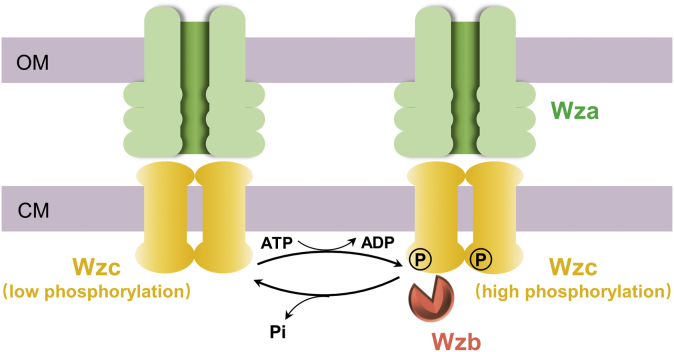
**Schematic presentation of the interactions of Wza, Wzb, and Wzc.** Wza forms an octamer for CPS export and Wzc forms an oligomer to interact with Wza. The production of CPS requires Wzc cycling between the high and low phosphorylation states, which is a combined result of the autokinase activity of Wzc and the phosphotase activity of Wzb. CM, cytoplasmic membrane; OM, outer membrane.

*Vv*Wzb belongs to the family of low-molecular-weight protein tyrosine phosphatase (LMWPTP). LMWPTPs are small (ca. 18 kDa), evolutionally conserved and important enzymes involved in various cellular processes of all kingdoms of life (20). They have been extensively studied over decades for the functional importance and druggability potential. So far, a number of structures of LMWPTPs from both eukaryotes and prokaryotes have been determined (21, 22, 23, 24, 25, 26, 27, 28, 29, 30). They share a common α/β fold; the active site crevice is composed of three characteristic loops, named the P-, D-, and W-loops, respectively. The P-loop bears the characteristic active-site motif “CXGNXCRS” (X can be any amino acid) with the first cysteine as the catalytic nucleophile; this loop is at the bottom of the active site crevice, extensively interacting with the phosphate group of substrate, thus named P-loop. The D-loop forms a wall of the active site crevice. It contains a catalytically important aspartate residue, which acts as a general acid/base, and also contains a tyrosine residue to stabilize the phenyl ring of the substrate. The third is a variable loop, forming another wall of the active site crevice and being responsible for substrate recognition. In the structures of eukaryotic and some prokaryotic LMWPTPs, an aromatic residue such as tryptophan is located at the variable loop to interact with the phenyl ring of the tyrosine phosphate. Thus, this loop is conveniently called W-loop. Nevertheless, the study on the structure of *E. coli* Wzb (*Ec*Wzb) uncovered another type of W-loop in prokaryotic LMWPTPs, which lacks this important aromatic residue and thus recognizes substrate in a different way (23). Noticeably, this study also showed that the W-loop type is a good marker to classify LMWPTPs. Indeed, the phylogenetic analysis confirmed that LMWPTPs can be divided into two categories, in agreement with the W-loop forms they have (23, 31). Hereafter, we would name the two categories of LMWPTPs as group I and II, prototyped by mammalian LMWPTPs and *Ec*Wzb, respectively. Interestingly, *Vv*Wzb is different from any structurally known LMWPTP by harboring a unique short sequence insertion in the W-loop, excluding *Vv*Wzb from either group (Fig. 2*A*). It is unclear if this could challenge the current classification paradigm of LMWPTP family. It also remains to know if the unique insertion would endow *Vv*Wzb with some new structural and enzymatic features.

**Figure 2:**
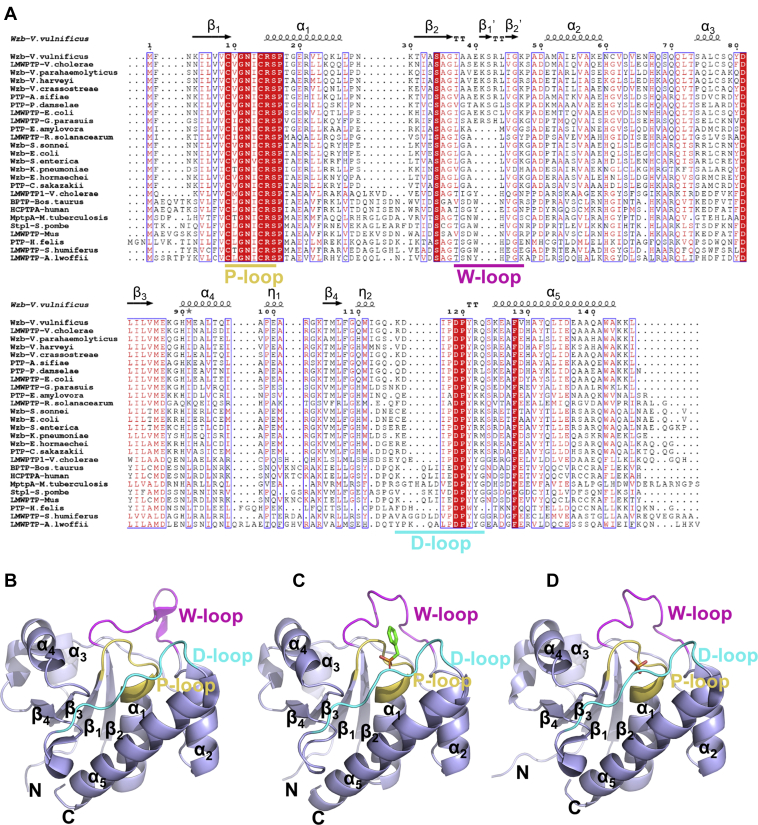
**Sequence alignment and the overall structures.***A*, sequence alignment of LMWPTPs. The conserved residues are highlighted in *red*. The secondary structures as well as the P-, D-, and W-loops are indicated. *B*−*D*, structures of free *Vv*Wzb, *Vv*Wzb-benzylphosphonate complex, and *Vv*Wzb^C9A^-phosphate complex, respectively. Secondary structures, the N- and C-termini are annotated. The P-, D-, and W-loops are colored in *yellow*, *blue*, and *magenta*, respectively. The ligands benzylphosphonate and phosphate are shown as *sticks*.

Herein, we report crystal structures of free *Vv*Wzb, *Vv*Wzb complexed with benzylphosphonate, and *Vv*Wzb^C9A^ complexed with phosphate. The structural and enzymatic analyses of *Vv*Wzb shed new insight into the classification and catalytic mechanism of the LMWPTP family. Our study also provides critical structural information for antivirulence drug design against *V. vulnificus*.

## Results

### Overall structures of VvWzb in free and ligand-bound forms

*Vv*Wzb crystallized in the space group P2_1_2_1_2_1_, with two protein molecules in one asymmetric unit. The two molecules formed a dimer in the crystal, with an interface area of about 900 Å^2^. However, this seems to be a crystallographic dimer, since *Vv*Wzb existed as a monomer in solution, indicated by gel filtration (Fig. S1). The structural model was built in good quality (Table 1). *Vv*Wzb adopts a typical LMWPTP α/β fold (Fig. 2*B*). In the middle is a twisted parallel β-sheet formed by four β-strands in the sequence of β_4_-β_3_-β_1_-β_2_; flanking the central β-sheet are five α-helices, with α_4_, α_3_ at one side and α_1_, α_2_, α_5_ at the other. The P-loop (between β_1_ and α_1_), D-loop (between β_4_ and α_5_), and W-loop (between β_2_ and α_2_) cluster together to form the active site crevice (Fig. 2*B*). Remarkably, the W-loop of *Vv*Wzb is longer than any known LMWPTP structure, due to a “^40^EKSR^43^” insertion (Fig. 2*A*). The W-loop adopts an extended conformation and protrudes from the protein body. The region harboring the unique insertion forms a small β-hairpin including two short β-strands (β_1_’ and β_2_’).

**Table 1:** Crystallographic statistics

Parameter	*Vv*Wzb	*Vv*Wzb-benzylphosphonate	*Vv*Wzb^C9A^-phosphate
Data collection statistics			
X-ray source	*BL19U1*	*BL19U1*	*BL18U1*
Wavelength (Å)	0.9788	0.9788	0.9792
Space group	P2_1_ 2_1_ 2_1_	P1	P2_1_ 2_1_ 2_1_
Unit-cell dimensions (Å or °)	*a* = 47.32, *b* = 48.74, *c* = 117.32	*a* = 44.11, *b* = 51.01, *c* = 68.59	*a* = 60.97, *b* = 68.30, *c* = 132.96
	*α* = 90.00, *β* = 90.00, *γ* = 90.00	*α* = 101.85, *β* = 95.56, *γ* = 103.79	*α* = 90.00, *β* = 90.00, *γ* = 90.00
Resolution (Å)	58.66−1.711 (1.740−1.711)	48.14−2.785 (2.833−2.785)	66.48−1.211 (1.232−1.211)
Unique reflections	29,957 (1506)	13,744 (689)	161,631 (7689)
Completeness (%)	99.5 (99.7)	97.9 (97.9)	95.7 (92.6)
Mean I/sigma(I)	19.2 (2.3)	16.4 (2.3)	17.9 (2.2)
Multiplicity	12.6 (12.9)	3.6 (3.6)	13.6 (13.9)
*R*_*merge*_ (%)	8.5 (118.8)	4.7 (56.8)	7.8 (107.7)
*R*_*measure*_ (%)	8.8 (123.8)	5.5 (66.6)	8.1 (111.8)
*R*_*pim*_ (%)	2.5 (34.3)	2.9 (34.5)	2.2 (29.7)
CC_1/2_ (%)	99.9 (78.8)	99.9 (81.1)	99.9 (93.3)
Refinement statistics			
Resolution range (Å)	58.66−1.71	48.14−2.79	29.72−1.211
*R*_*work*_/*R*_*free*_ (%)^a^	19.9/22.3	18.6/22.6	14.3/16.9
Modeled residues	A: −1 to 146 B: −1 to 146	A: 1−146 B: 0−146 C: 1−146 D: 1−146	A: 1−146 B: 1−146 C: −2 to 146 D: −2 to 146
Ligands	2 Cl^−^	4 benzylphosphonate	4 phosphate ions, 1 glycerol
Water molecules	270	None	679
rmsd bond lengths (Å)	0.010	0.010	0.002
rmsd bond angles (°)	1.02	1.12	0.59
Ramachandran outliers	0	0	0
PDB code	7DHD	7DHE	7DHF

Values in parentheses represent the highest resolution shell.

a ∼5% of the reflections were selected randomly for calculating *R*_*free*_.

**Figure 3:**
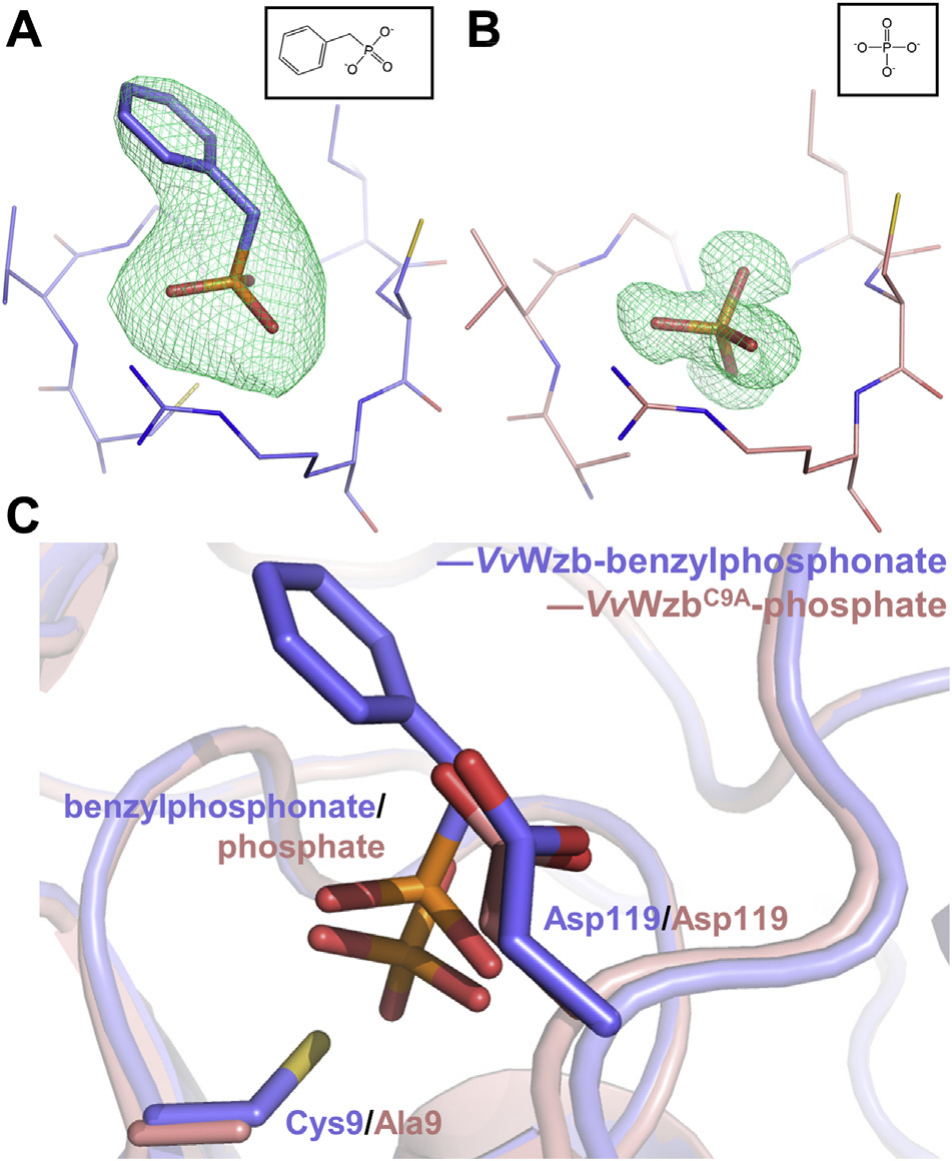
**Ligands in the complex structures.***A*, benzylphosphonate in *Vv*Wzb-benzylphosphonate structure. *B*, phosphate in *Vv*Wzb^C9A^-phosphate structure. The representative refined omit Fo-Fc maps contoured at 2.5 σ levels were shown as *green meshes*. The chemical structures of the ligands are displayed as *insets*. *C*, ligand-binding differences in *Vv*Wzb-benzylphosphonate and *Vv*Wzb^C9A^-phosphate structures. Benzylphosphonate, phosphate, the residues Cys9/Ala9 and Asp119 are shown as *sticks* and indicated.

In the presence of benzylphosphonate, a compound that mimics the phenylphosphate moiety of the substrate phosphotyrosine, *Vv*Wzb and *Vv*Wzb^C9A^ crystallized in space groups P1 and P2_1_2_1_2_1_, respectively. Both crystals contained four protein molecules in one asymmetric unit. The dimeric form observed in the free *Vv*Wzb crystal did not show up again, and instead two adjacent molecules were covalently crosslinked *via* a disulphide bond formed by the Cys61 residues of both molecules. We were able to model one benzylphosphonate molecule (with occupancy of 1 and B-factor of 82.8, 142.8, 109.6, or 89.9 Å^2^) for each of the four protein chains in the wild-type (WT) structure (Figs. 2*C* and 3*A*). However, only phosphate ions were identified in *Vv*Wzb^C9A^ structure, judged by the unambiguous electron density (Figs. 2*D* and 3*B*). The source of the phosphate ion was not defined, presumably from benzylphosphonate degradation or contamination. The overall structures of *Vv*Wzb-benzylphosphonate and *Vv*Wzb^C9A^-phosphate are similar to that of free *Vv*Wzb, except that the W-loop underwent significant conformational changes to fold toward the active site in both structures (Fig. 2, *C* and *D*).

### The active site crevice of VvWzb

**Figure 4:**
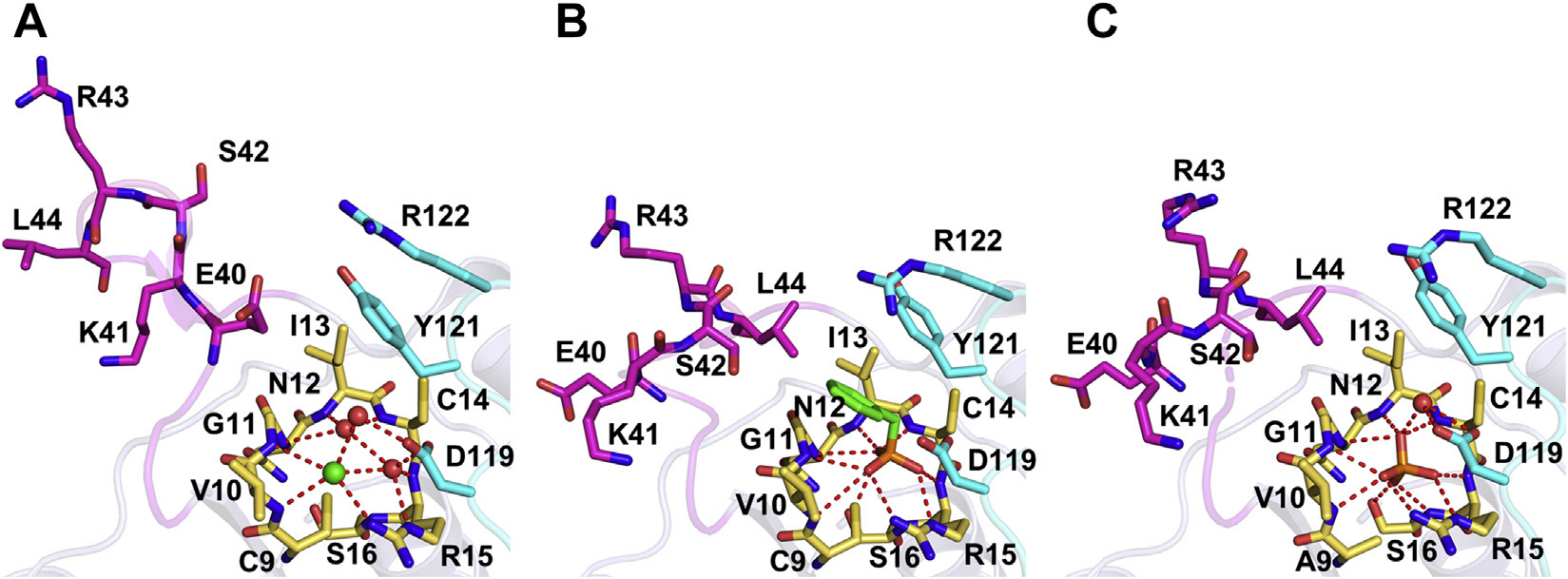
**Close-up of the active site regions.***A*, free *Vv*Wzb. *B*, *Vv*Wzb-benzylphosphonate complex. *C*, *Vv*Wzb^C9A^-phosphate complex. The P-, D-, and W-loops are colored in *yellow*, *cyan*, and *magenta*, respectively. Key residues and the ligands are represented in *sticks*; chloride ion and water molecules are represented by *green* and *red spheres*, respectively. The hydrogen bonds are shown as *red dotted lines*.

Like other LMWPTPs, the active site crevice of free *Vv*Wzb is composed of the P-loop (residues Cys9−Ser16), D-loop (residues Lys115−Gln123), and W-loop (residues Ile37−Lys47) (Figs. 2*A* and 4*A*). The P-loop, containing the catalytic motif ^9^CVGNICRS^16^, with Cys9 as the catalytic nucleophile, forms the bottom part of the crevice. The residues Asp119, Tyr121, and Arg122 of the D-loop are stacked together above the P-loop to form a wall of the crevice, and the W-loop forms the opposite wall. The W-loop of *Vv*Wzb, which is longer than any known LMWPTP by harboring a four-residue insert (^40^EKSR^43^), bulges out, with residues Ser42, Arg43, and Leu44 located at the tip region and residue Glu40 pointing toward the active site. Compared with the D-loop wall, which is relatively orthogonal to the P-loop bottom, the W-loop wall seems to be laid flat. In such a configuration, the active site crevice of free *Vv*Wzb is wide and shallow, being readily accessible for the substrate (Fig. S2). Due to the presence of Glu40, the W-loop wall region of the crevice is negatively charged. In the absence of ligand, the supposed phosphate-binding site is occupied by a chloride ion and a few water molecules; they interact with the P- and D-loop residues *via* extensive hydrogen bonding (Fig. 4*A*).

In *Vv*Wzb-benzylphosphonate complex, the substrate-mimicking benzylphosphonate is located at the bottom of the active site crevice. Its phosphonate group forms an extensive hydrogen-bonding network with the P-loop, including the main chain amide groups of the residues Val10, Gly11, Asn12, Ile13, Cys14, and Arg15, as well as the sulfhydryl group of Cys9 and the side-chain guanidine group of Arg15. The benzyl group of benzylphosphonate is flanked by residues Val10 of the P-loop and Tyr121 of the D-loop and further stabilized by Leu44 of the W-loop and Ile13 of the P-loop *via* hydrophobic interactions (Fig. 4*B*). Compared with the free structure, the W-loop undergoes a dramatic conformational rearrangement from an open to a closed conformation. Consequently, Glu40 is no longer oriented toward the active site cavity but turns outside. Instead, side chains of Ser42 and Leu44 turn to face the active site, forming the W-loop wall. In addition, side chains of Asp119 and Tyr121 of the D-loop also made small local conformational changes to interact with the ligand (Fig. 4, *A* and *B*). All these conformational changes significantly reconfigure the active site crevice, which is narrower and deeper in shape as well as more positively charged compared with that of free *Vv*Wzb (Fig. S2).

*Vv*Wzb^C9A^-phosphate shares a similar active site structure with the *Vv*Wzb-benzylphosphonate complex. Mutating Cys9 to an alanine residue that has a short and uncharged side chain allowed the phosphate ion to burry deeper in the active site than the phosphonate moiety of benzylphosphonate (Fig. 3*C*). As a result, the hydrogen bonds between the phosphate ion and the P-loop residues are generally shorter than those observed in *Vv*Wzb-benzylphosphonate, suggesting a stronger binding. Among these interactions, two hydrogen bonds contributed by the main-chain NH and the side-chain hydroxyl group of Ser16 are not observed in the *Vv*Wzb-benzylphosphonate complex. Overall, the structure of *Vv*Wzb^C9A^-phosphate mimics that of the phosphoryl-enzyme intermediate, judged by the position and orientation of the phosphate ion (28, 32). At the distance of about 3.5 Å to the phosphorous atom, we identified a water molecule that forms a hydrogen bond with Asp119 (Fig. 4*C*). This water molecule is assumed to be activated by Asp119 and functions as a nucleophile in the dephosphorylation reaction of the phosphoryl-enzyme intermediate. The phosphate ion does not interact directly with the D- and W-loop residues; however, the conformations of these loops are similar to those observed in *Vv*Wzb-benzylphosphonate complex, suggesting that the phosphate ion alone is sufficient to induce the conformational changes of the D- and W-loops. The electrostatically repelling between Glu40 and the phosphate ion is supposed to be an important driving force for the structural rearrangement of the W-loop. Overall, the shape and charge distribution of the active site crevice are similar to that in the *Vv*Wzb-benzylphosphonate complex.

### Structural comparison with other LMWPTPs

As expected, a number of known LMWPTPs were identified by the Dali server to be structurally similar to *Vv*Wzb. Generally, *Vv*Wzb shares more similarity in structure and sequence with the *Ec*Wzb-prototyped group II LMWPTPs (rmsd of aligned Cα atoms of 0.8–0.9 Å^2^, sequence identity of 52–58%) than the group I LMWPTPs prototyped by mammalian LMWPTPs (rmsd of aligned Cα atoms of 1.4–1.9 Å^2^, sequence identity of 22–37%). In addition, arsenate reductases were also identified to be structurally similar to *Vv*Wzb (rmsd of aligned Cα atoms of about 2.1 Å^2^, sequence identity of about 23%), supporting the proposal that LMWPTP originated from prokaryotic arsenate reductase (20).

**Figure 5:**
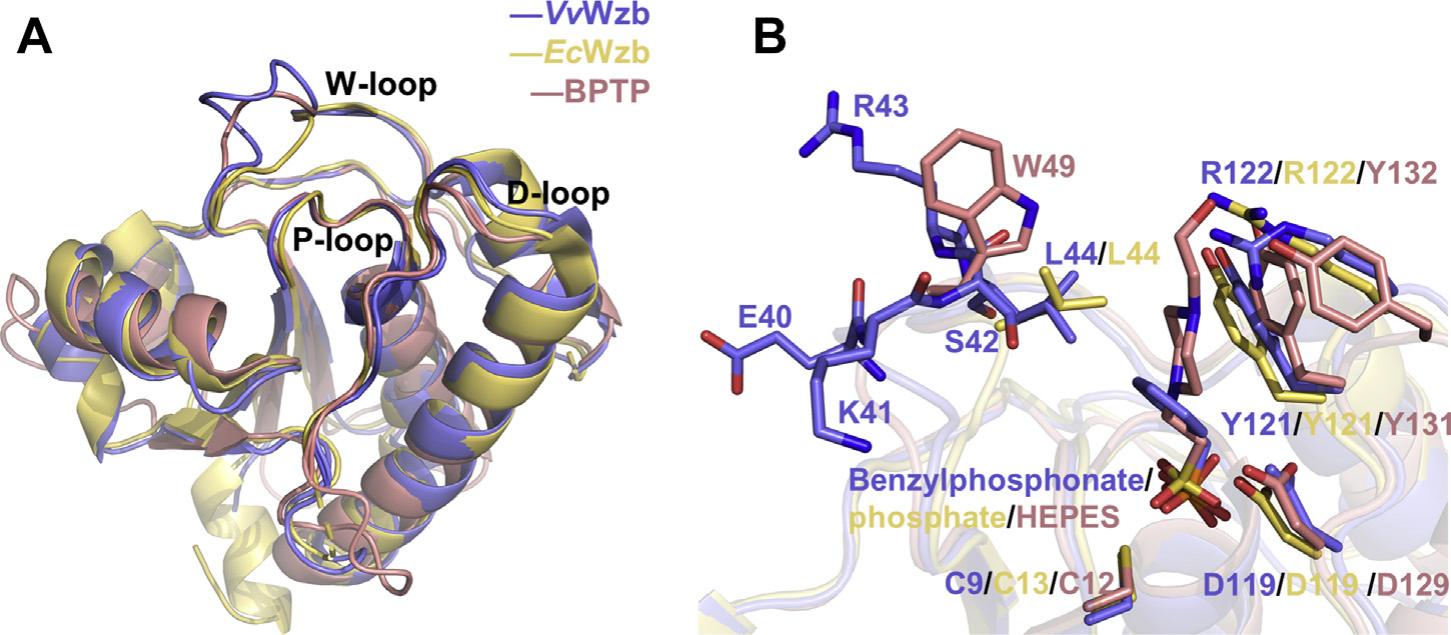
**Structural comparison of *Vv*Wzb, *Ec*Wzb, and BPTP.***A*, superimposition of the overall structures. The P-, D-, and W-loops are annotated. *B*, close-up of the active site region. The ligands and key residues are shown as *sticks* in their respective colors.

We chose two typical LMWPTPs, group II prototype *Ec*Wzb (PDB ID: 2WJA, sequence identity of 57% to *Vv*Wzb) and group I prototype BPTP (PDB ID: 1DG9, sequence identity of 29% to *Vv*Wzb) for comparison to understand the similarity and difference between *Vv*Wzb and other LMWPTPs (Fig. 5). All structures are in ligand-bound forms. Overall, structures of *Vv*Wzb, *Ec*Wzb, and BPTP could be well aligned for the regulatory secondary structures, with relatively large variations mainly occurring in the loop regions (Fig. 5*A*). We focused on the comparison of the active site crevices, which directly determine the substrate specificity and catalytic power. The P-loops of *Vv*Wzb, *Ec*Wzb, and BPTP contain the motifs “CVGNICRS”, “CTGNICRS,” and “CLGNICRS”, respectively. Despite the sequence variation at the second position of the catalytic motif, the P-loops of all structures are well superposed in space, in agreement with the conserved phosphate-binding function of this loop. Regarding the D-loop, *Vv*Wzb shares more similarity with *Ec*Wzb than with BPTP. *Vv*Wzb and *Ec*Wzb contain one tyrosine residue followed by an arginine residue in the D-loop, while BPTP contains two tyrosine residues (Tyr131 and Tyr132) (Fig. 5*B*). These two adjacent tyrosine residues are generally found in group I LMWPTPs, endowing their D-loops with a phosphorylation-mediated regulatory function (33). Phosphorylation on the D-loop has also been observed in group II LMWPTP (34) and could occur in *Vv*Wzb as well, due to the similar D-loop compositions. In group I LMWPTPs, the D-loop can undergo conformational changes upon ligand binding (35), but this is not observed in group II LMWPTPs. For *Vv*Wzb, there were only certain local conformational changes in the D-loop upon ligand binding. Remarkably, the most varied region of all three enzymes is located at the W-loop (Fig. 5*B*). The W-loop of *Ec*Wzb is the shortest among the three; a leucine (Leu44) is supposed to interact hydrophobically with the substrate. BPTP contains a tryptophan (Trp49) in the W-loop, whose side chain can interact hydrophobically with the phenyl ring of the substrate. In *Vv*Wzb, the four-residue insertion is partially overlapped with the side chain of Trp49 of BPTP. These inserted residues do not show direct interactions with the ligand due to a long distance between them. Instead, a leucine residue in *Vv*Wzb (Leu44) corresponding to *Ec*Wzb Leu44 may provide hydrophobic interaction with the phenyl ring of the ligand. The specific configuration of these loops makes the active site crevice of *Vv*Wzb different from those of previously studied LMWPTPs in shape and charge (Fig. S2). In shape, the active site crevices of group II LMWPTPs are relatively small and shallow, while those of group I LMWPTPs appear large and deep, showing a higher W-loop wall, which is attributed to their characteristic aromatic residue in the W-loop. The active site crevice of *Vv*Wzb in the ligand-free state is small and shallow but becomes larger and deeper in the ligand-bound state, resembling those of group I LMWPTPs. Regarding charge, the active site crevices of all LMWPTPs share a positively charged bottom, attributed to the conserved Arg residue of the P-loop. Group I LMWPTPs prefer relatively neutral walls for the active site crevices, while group II tends to have more positive ones. In comparison, the active site crevice of *Vv*Wzb is the most positively charged, largely due to the extra residue Lys41 in its W-loop wall. Assumingly, the distinct shapes and charge patterns of the active site crevices would exert profound effects on substrate recognition and catalysis for LMWPTPs.

### Enzymatic characterization of VvWzb and its mutants

*Vv*Wzb exhibited clear phosphatase activity in the assay using para-nitrophenyl phosphate (pNPP) as substrate (Table 2, Fig. S3). Compared with *Ec*Wzb, *Vv*Wzb has a remarkably higher *k*_cat_ and a comparable *K*_*m*_. Overall, the catalytic efficiency (*k*_cat_/*K*_*m*_) of *Vv*Wzb is approximately eightfolds of that of *Ec*Wzb. To understand the roles of individual residues in catalysis, we made several mutants and measured their activities. Based on structural analysis and previous knowledge of LMWPTPs, residues in the P- and D-loops are supposed to play primary roles in catalysis of *Vv*Wzb: Cys9 is a nucleophile; Asp119 serves as the general acid/base in the catalysis; Arg15 is crucial to interact with the phosphate group to stabilize the transition state. Mutating these individual residues of *Vv*Wzb to alanine (*Vv*Wzb^C9A^, *Vv*Wzb^R15A^, and *Vv*Wzb^D119A^) almost abolished the enzymatic activity, conforming their essential roles in catalysis.

**Table 2:** Enzyme kinetics data

Protein	*k*_*cat*_ (s^−1^)	*K*_*m*_ (mM)	*k*_*cat*_/*K*_*m*_ (mM^−1^∗s^−1^)	Relative activity (%)
*Vv*Wzb	25.10 ± 0.24	3.94 ± 0.12	6.37	100
*Vv*Wzb^E40-R43del^	6.00 ± 0.20	14.32 ± 1.07	0.42	6.58
*Vv*Wzb^E40-R43delinsW^	25.56 ± 0.23	7.90 ± 0.63	3.24	50.78
*Vv*Wzb^K41A^	19.68 ± 1.16	6.03 ± 1.01	3.26	51.08
*Vv*Wzb^K41E^	26.60 ± 0.64	7.03 ± 0.49	3.78	59.42
*Vv*Wzb^S42A^	25.94 ± 0.20	4.21 ± 0.22	6.16	96.71
*Vv*Wzb^S42E^	32.08 ± 1.19	6.74 ± 0.44	4.76	74.78
*Vv*Wzb^E40-R43delinsAAAA^	31.02 ± 0.86	8.47 ± 0.27	3.66	57.52
*Ec*Wzb	2.84 ± 0.10	3.48 ± 0.24	0.82	100 (12.87)^a^
*Ec*Wzb^A39_L40insEKSR^	9.34 ± 0.10	2.53 ± 0.03	3.69	450 (57.93)^a^
*Ec*Wzb^A39_L40insAAAA^	6.09 ± 0.09	2.05 ± 0.10	2.97	362 (46.63)^a^

The values of *k*_*cat*_ and *K*_*m*_ are expressed as mean ± SEM of three independent experiments. Relative activity is defined as the catalytic efficiency (*k*_*cat*_/*K*_*m*_) divided by that of WT. The kinetics of *Vv*Wzb^C9A^, *Vv*Wzb^R15A^, and *Vv*Wzb^D119A^ were not determined for their very low activity.

a The values in the parenthesis indicate the relative activity to that of WT *Vv*Wzb.

Compared with other LMWPTPs, the W-loop of *Vv*Wzb is special by containing a unique “^40^EKSR^43^” insertion (Fig. 2*A*). To probe the function of this insertion, we first deleted the four residues to mimic the W-loop of group II LMWPTPs. In comparison with the WT, the deletion mutant *Vv*Wzb ^E40-R43del^ exhibited about fourfolds decrease for *k*_*cat*_, while the substrate-binding affinity was also significantly lowered judged by the increased *K*_*m*_. Altogether, the deletion mutant showed a significant decrease in the catalytic efficiency up to 15 folds (Table 2, Fig. S3). We also replaced these four residues with a tryptophan to mimic group I LMWPTPs. The tryptophan variant *Vv*Wzb ^E40-R43delinsW^ showed a similar *k*_*cat*_ to that of WT, but the substrate-binding affinity was lower than WT judged by the twofold *K*_*m*_ value. Overall, it showed a half of the catalytic efficiency of WT, which is much higher than the deletion mutant. These results indicate that the four-residue insertion “^40^EKSR^43^” is required for an optimal activity of *Vv*Wzb, and the enhancing effect is even higher than a single tryptophan residue. We were able to find similar four-residue insertion in the W-loops of many *Vv*Wzb homologs, showing that Lys41 and Ser42 are conserved in the new type of W-loop (Fig. 2*A*). These two residues face toward the active site, possibly affecting ligand binding (Fig. 4*B*). Therefore, we tested the contributions of Lys41 and Ser42 to catalysis by mutagenesis. The mutants *Vv*Wzb^K41A^, *Vv*Wzb^K41E^, and *Vv*Wzb^S42E^ maintained about half enzymatic activity of WT, and *Vv*Wzb^S42A^ even showed an activity of 97% of that of WT. Apparently, the activity is not dependent on the side chain types at these two positions. To further test the sequence-independency hypothesis, we replaced “^40^EKSR^43^” with four alanine residues. Again, the *Vv*Wzb^E40-R43delinsAAAA^ mutant showed more than half enzymatic activity of WT, with a slightly higher *k*_*cat*_ and a doubled value of *K*_*m*_. This result confirmed that the residue type is not critical for the activity enhancing effect of the insertion. Probably, a local structure derived from a simple four-residue skeleton is sufficient for the enhancing effect. Taken together, the four-residue insertion in the W-loop of *Vv*Wzb endowed the enzyme with a higher enzymatic activity than null or tryptophan residue at the same position of the W-loop. The enhancing effect is not determined by the sequence specificity of the insertion.

We wondered if this enhancing effect of the four-residue insertion can also be applied to other LMWPTPs. Therefore, we performed a gain-of-function experiment by inserting the four residues in the corresponding position of *Ec*Wzb. Both mutants *Ec*Wzb^A39-L40insEKSR^ and *Ec*Wzb^A39-L40insAAAA^ showed a significant increase (∼fivefolds/fourfolds) in catalytic efficiency (Table 2, Fig. S3). The enhancing effect was mainly reflected by the increase in *k*_*cat*_, which is about three or two times of that of the WT. For both mutants, the *K*_*m*_ values are slightly lowered, indicating a better substrate-binding affinity. These results suggest that a four-residue insertion at the W-loop can be a general activity enhancing element for the LMWPTP fold.

**Figure 6:**
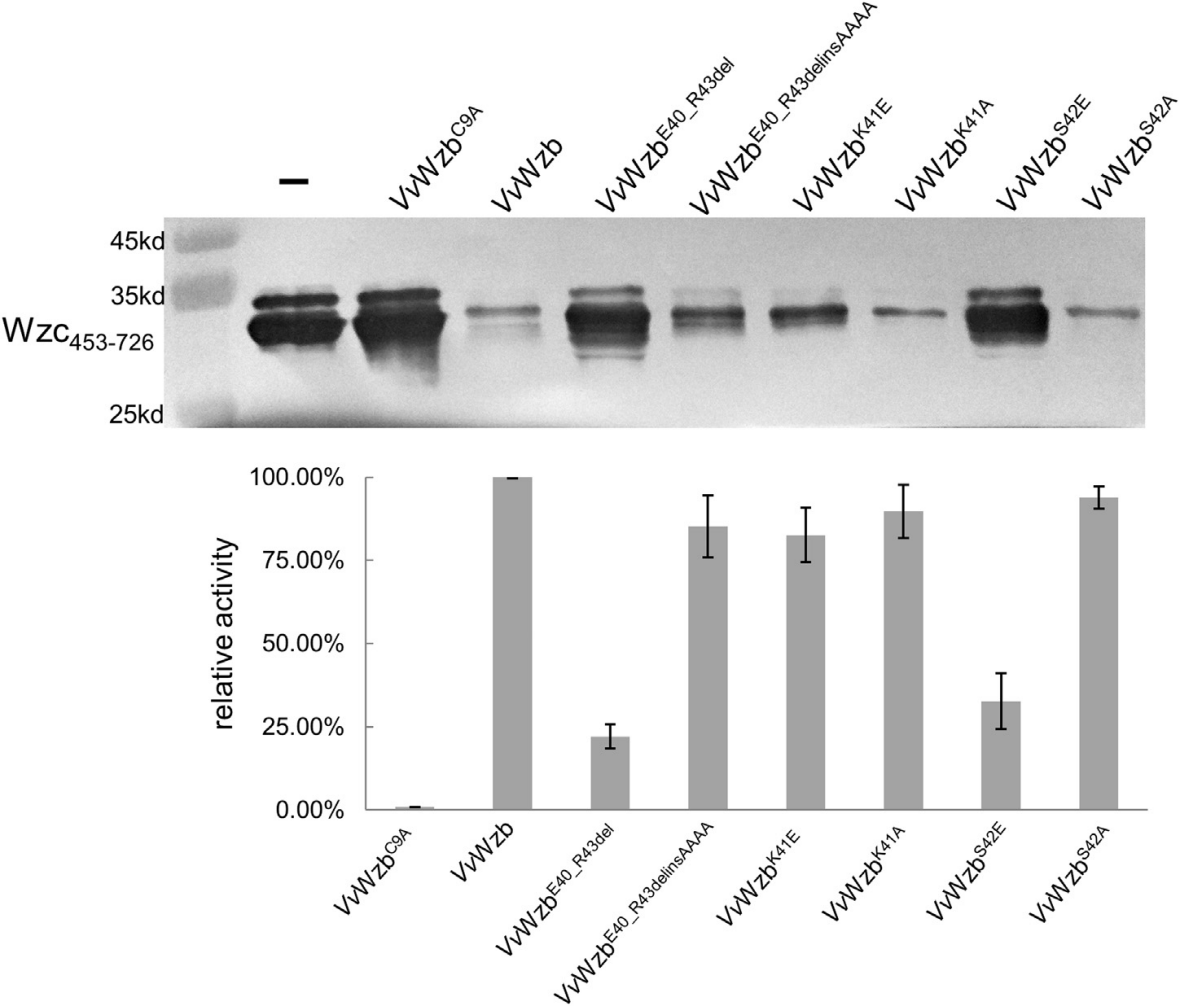
**Dephosphorylation of *Vv*Wzc**_**453−726**_**by *Vv*Wzb and its mutants.** The *upper panel* shows a representative western blot of *Vv*Wzc_453−726_ stained by anti-phosphotyrosine antibody after incubation with *Vv*Wzb and the mutants. The *lower panel* shows the relative enzymatic activity of these enzymes. The error bars stand for the standard deviation values (n = 3).

All aforementioned results are based on the enzymatic assays using pNPP as the substrate. We wondered if these results can be verified when using a physiological substrate. We purified *Vv*Wzc_453−726_, the Y-cluster of which serves as the natural substrate of *Vv*Wzb. The recombinant *Vv*Wzc_453–726_ showed two bands that were stained by the anti-phosphotyrosine antibody, probably representing two phosphorylation states. After incubation with WT *Vv*Wzb and the mutants, we observed dephosphorylation of *Vv*Wzc_453−726_ to various extents. The WT dephosphorylated *Vv*Wzc_453−726_ in a short time period, but did not lead to a complete dephosphorylation. The mutants *Vv*Wzb^K41A^, *Vv*Wzb^K41E^, *Vv*Wzb^S42A^, and *Vv*Wzb^E40-R43delinsAAAA^ showed 82% to 94% dephosphorylating activities of the WT, while the activity of *Vv*Wzb ^E40-R43del^ is poor, with 22% activity retained (Fig. 6). In general, the relative activities of these mutants showed the same trend as that observed in the pNPP assays. Interestingly, the mutant *Vv*Wzb^S42E^ showed inconsistent results in the two assays. In the pNPP assay, this mutant showed a high enzymatic activity, similar to that of other point mutations (Table 2, Fig. S3). However, its activity (33% activity of WT) was much lower than other point mutants but comparable with that of the deletion mutant, when using *Vv*Wzc_453−726_ as substrate (Fig. 6). This phenomenon indicates that an unfavourable interaction between the W-loop of *Vv*Wzb^S42E^ and *Vv*Wzc_453−726_ occurred, resulting in enzymatic activity loss.

## Discussion

Herein, we report the crystal structures of *Vv*Wzb in free and ligand-bound forms. *Vv*Wzb contains an unusual W-loop with a four-residue insertion, which is required for the optimal activity. This is the first report to identify and characterize such a novel W-loop in the LMWPTP family.

Remarkably, the unique insertion in the W-loop endows *Vv*Wzb with unprecedented structural and functional features in the LMWPTP family. The P- and D-loop regions of *Vv*Wzb resemble those of known LMWPTPs, but its W-loop is longer than any other known LMWPTPs for containing a unique four-reside insertion. The W-loop of *Vv*Wzb exhibited an extended conformation in ligand-free state but underwent dramatic conformational changes upon ligand binding, which reconfigured the shape and charges of the active site crevice. Conformational changes in the W-loop were reported in group I LMWPTPs before, but they were only small changes on the orientation of the tryptophan side chain (35). This is the first time to observe such a large conformational change of the W-loop in the LMWPTP family. Importantly, the unique four-residue insertion in the W-loop of *Vv*Wzb showed functional importance. The insertion not only was required for an optimal activity of *Vv*Wzb but also enhanced the activity of *Ec*Wzb once incorporated into the latter at the corresponding position. Roughly, the enhancing effect of the four-residue insertion is comparable with that of a tryptophan residue. The exact mechanism of the enhancement remains to be understood, but it is obviously different from that of an aromatic residue, which stabilizes the phenyl ring of the substrate to gain higher catalytic power (23). Surprisingly, substituting the insertion to a simple four-alanine patch is sufficient to maintain a high enzymatic activity. It appears that the space effect instead of the sequence specificity of the insertion makes the major contribution to activity enhancement. Interestingly, we also observed that the *Vv*Wzb^S42E^ mutant showed a largely reduced enzymatic activity when using *Vv*Wzc_453−726_ as substrate, even though its activity was comparable with that of WT when using pNPP as substrate. In the complex structures, Ser42 points to the active site pocket. When substituting to Glu, the longer side chain may hinder the localization of the Y-cluster of *Vv*Wzc_453−726_, whereas this is less hazardous for the smaller substrate pNPP. Alternatively, the W-loop of *Vv*Wzb^S42E^ interacts with *Vv*Wzc_453−726_ and thus is not able to take the correct conformation for catalysis. Either explanation inspires a hypothesis of regulation mechanism. It is known that glutamic acid residue is a mimic of phosphoserine. In fact, Ser42 is located at the tip region of the W-loop as shown in the ligand-free *Vv*Wzb structure and thus easily accessible by a kinase. It is plausible that Ser42 of *Vv*Wzb can be phosphorylated and thus regulate the enzymatic activity. The hypothesis is not unreasonable, considering that phosphorylation-mediated regulation has been reported for LMWPTPs (36). This might explain why the serine residue is conserved in this new type of W-loop (Fig. 2*A*).

**Figure 7:**
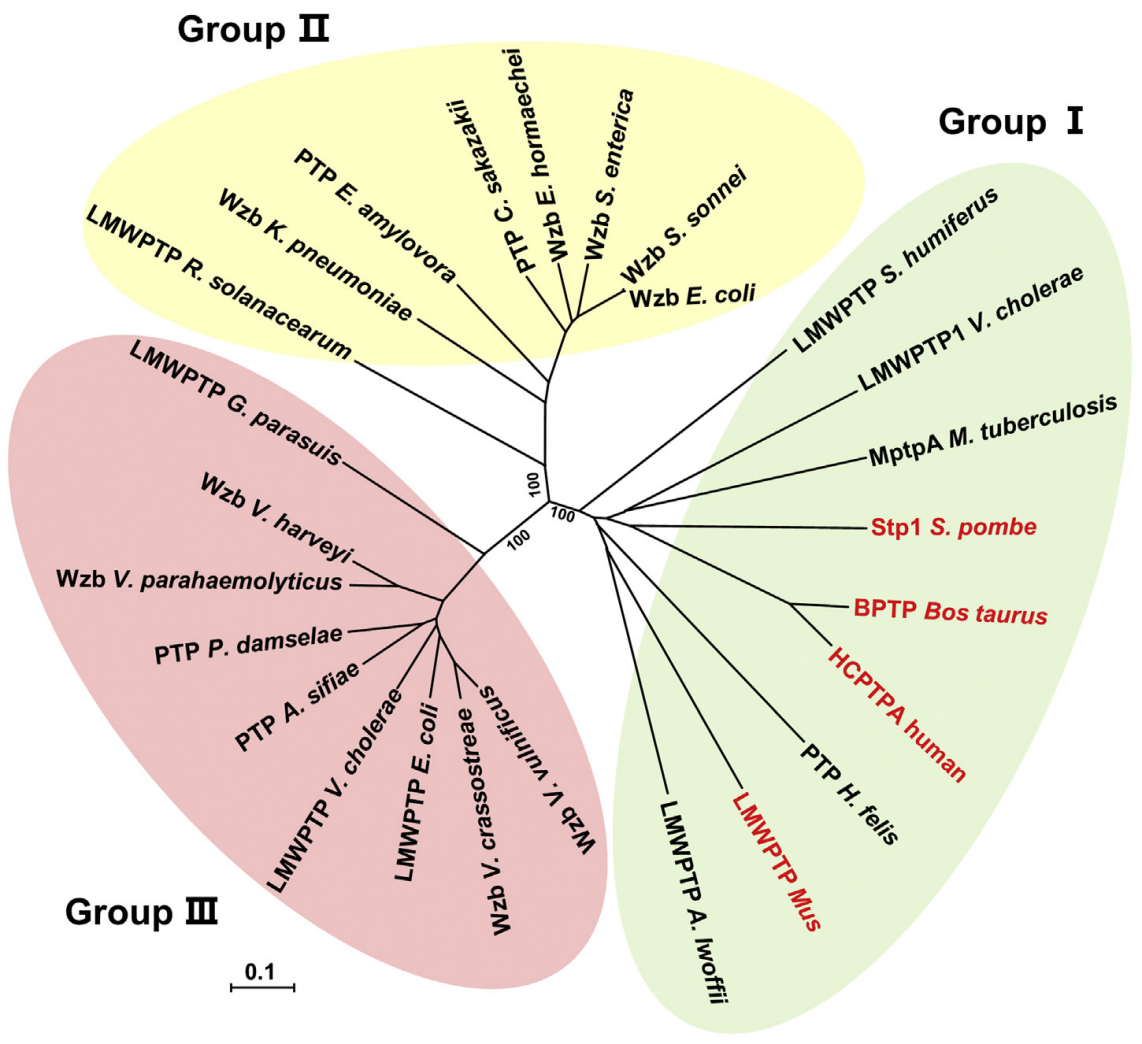
**Phylogenetic analysis of the LMWPTP family.** LMWPTPs from prokaryotes and eukaryotes are shown in *black* and *red words*, respectively.

Our study also raises a request to update the current classification of LMWPTP family. In the last decades, a number of LMWPTP structures have been determined and there are only two types of W-loop identified (23). The W-loop containing a characteristic aromatic residue has been identified in the majority of the LMWPTP structures, including BPTP (PDB ID: 1DG9), HCPTPA (PDB ID: 5PNT), HCPTPB (PDB ID: 1XWW), and *Vc*LMWPTP1(PDB ID: 4LRQ) (25, 26, 32, 37). The other type of W-loop lacks this aromatic residue, found in structures such as *Ec*Wzb (PDB ID: 2WJA), *Ea*AmsI (PDB ID: 4D74), and *Vc*LMWPTP2 (PDB ID: 5Z3M) (29, 38, 39). The two types of W-loop employ distinct substrate recognition mechanisms (23). Interestingly, phylogenetic analysis has divided LMWPTPs into two groups, which is parallel to their W-loop types (23, 31). This supports the role of W-loop as an evolutional marker in classification of the LMWPTP family. Apparently, *Vv*Wzb employs a new type of W-loop and is outside of either group. Moreover, this type of W-loop is not limited to *V. vulnificus*, but spreads widely in the γ-*proteobacteria* class, including the families of Vibrionaceae, Enterobacteriaceae, and Pasteurellaceae. Further, we carried out a phylogenetic analysis that grouped the LMWPTPs into three clades, including the previously known two groups and the third one containing *Vv*Wzb and its homologs (Fig. 7). In agreement with previous observation, the phylogenetic grouping is obviously in parallel to the W-loop types. Taken the W-loop type and phylogenetic relationship together, we suggest to reclassify LMWPTPs into three groups: I to III, prototyped by mammalian LMWPTPs, *Ec*Wzb, and *Vv*Wzb, respectively. It is known that indels, referring to short insertions and deletions in the protein sequence, are a major force to drive protein evolution to gain new structural folds, new functionalities, and new regulations (40, 41, 42). W-loop diversity in LMWPTPs is an excellent example in this context. Based on previous results and our study, it is clear that indel variations in the W-loop endow LMWPTPs with specific structural, dynamic, and mechanical features. The coincidence between the W-loop types and phylogenetic relationship also demonstrates that indel occurring at the W-loop plays important roles in driving the evolution and diversity of LMWPTP family.

Characterization of the protein tyrosine phosphorylation system in prokaryotes is still at infancy in comparison with that in eukaryotes (43). LMWPTPs are important components in both prokaryotic and eukaryotic tyrosine phosphorylation systems. Here, we notice a difference in their LMWPTP diversities. Based on the phylogenetic tree, eukaryotes use only group I LMWPTPs (Fig. 7). This sole type of LMWPTPs can dephosphorylate an array of protein substrates in various cellular processes (1). In contrast, prokaryotes employ all three groups of LMWPTPs. It is not unusual that one species contains two groups of LMWPTPs, being a combination of any two groups. For example, *V. vulnificus* contains group I (protein ID: WP_130193014.1) and III (protein ID: WP_017420711.1) LMWPTPs. Even, some species contain all three groups, exemplified by *E. coli* (with protein IDs: WP_153671227.1, WP_052935621, and AYN88831.1) and *Vibrio cholerae* (with protein IDs: WP_001909008.1, WP_001880745.1, and WP_114808776.1). It is also interesting to find that a similar biochemical process in different species can employ different groups of LMWPTPs. For instance, the above-mentioned *Ec*Wzb (group II) and *Vv*Wzb (group III) are all related to polysaccharide synthesis (18, 44). Since W-loop plays an important role in substrate recognition and ligand regulation (25), using diverse LMWPTPs with all types of W-loops may endow prokaryotes with certain advantages in substrate spectrum and regulation to adapt to their large variety of cellular processes. It is known that most prokaryotes use BY-kinases, with no homologs in eukaryotes (45). Now, from both the aspects of kinase and phosphatase, it is clearer that prokaryotic tyrosine phosphorylation system is not analogous to that of eukaryotes, but has its own unique characteristics. The evolutional relationship between prokaryotic and eukaryotic tyrosine phosphorylation systems is an interesting question for future study.

Finally, our study also provides an opportunity to develop antivirulence agents against *V. vulnificus*. LMWPTP family members have been proposed in recent years as promising drug targets for human diseases, including cancer, diabetes, and infectious diseases, with many potential inhibitors developed (46, 47, 48). Obviously, inhibiting *Vv*Wzb would damage the CPS production of *V. vulnificus* and thus render the bacterium to the biocidal attack of the host immune system, making *Vv*Wzb an excellent antivirulence target. Our structural analysis of *Vv*Wzb in free and ligand-bound forms has described a clear profile of its ligand-binding pocket, facilitating inhibitor development through a structure-based approach. The chemical compounds developed for other LMWPTPs can be a good start to design *Vv*Wzb inhibitors. More importantly, the unique insertion in the W-loop endows *Vv*Wzb with an active site pocket distinct from that of the LMWPTPs of the eukaryotic hosts and most microbiota bacteria, giving a good opportunity to develop pathogen-specific inhibitors. To note, conformational changes of the W-loop of *Vv*Wzb may be induced by inhibitor binding, which should be taken into account during inhibitor developing process.

## Experimental procedures

### Gene cloning and protein production

The DNA sequence encoding *Vv*Wzb was amplified from the genomic DNA of *V. vulnificus* ATCC 27562 by standard PCR method using primers (forward, 5′-AGTCCATGGGCTTTAACAAAATCTTAGTCGTTTG-3′; reverse, 5′-CCGCTCGAGCTACAATTTTTTTGCCCAG-3′). The PCR product was inserted into the vector pETM11 (EMBL) using NcoI and XhoI sites. The product from this construct would be composed of an N-terminal 6×His tag, a tobacco etch virus (TEV) cleavage site, and the *Vv*Wzb coding sequence.

The sequence-verified gene construct was transformed into the *E. coli* C43 (DE3) strain. The strain was cultivated in Luria–Bertani (LB) broth medium containing 50 μg/ml kanamycin. When the OD_600_ value reached about 0.8, isopropyl-β-D-thiogalactopyranoside (IPTG) at a final concentration of 200 μM was added to induce the overexpression of the target protein. The induced cells were further cultured at 16 °C for 14 h with vigorous shaking. Subsequently, the cells were harvested by centrifugation at 6000*g* for 10 min. The cell pellets were resuspended in the binding buffer containing 50 mM Tris--HCl pH 8.0, 150 mM NaCl, then lysed by high-pressure cell disruptor. The lysate was centrifuged at 15,000*g* for 1 h at 4 °C, and the supernatant was loaded onto a nickel-chelating Sepharose affinity chromatography column (BBI). The purified protein was eluted with the buffer containing 50 mM Tris-HCl pH 8.0, 150 mM NaCl, 250 mM imidazole. Then the protein solution was changed to 50 mM TrisHCl pH 8.0, 150 mM NaCl using a PD-10 column (GE Healthcare) for TEV (with His-tag) digestion. The tag-uncleaved protein and TEV were removed by reloading the solution onto the nickel-chelating Sepharose affinity chromatography column. The flow-through containing untagged protein was collected, concentrated, and purified using gel filtration on a HiLoad 16/600 Superdex 75 column (GE Healthcare) equilibrated with the buffer containing 10 mM HEPES pH 7.5, 150 mM NaCl, 1 mM DTT. The purified protein was concentrated to approximately 20 mg/ml as determined by the absorbance at 280 nm, then used immediately or freshly frozen at −80 °C for later use.

*Ec*Wzb gene was cloned from the *E. coli* strain K12 and incorporated into pETM11 using ClonExpressII One Step Cloning Kit (Vazyme). The mutant genes in this study were prepared by overlapping PCR and inserted into pETM11 vector using restriction-ligation method or ClonExpressII One Step Cloning Kit (Vazyme). The overexpression and purification procedures were similar to those of WT *Vv*Wzb.

To prepare the protein substrate for *Vv*Wzb, the DNA sequence encoding the C-terminal amino acid residues 453 to 726 of Wzc of *V. vulnificus* ATCC 27562 (*Vv*Wzc_453−726_) was amplified by primer (forward, 5′-TTTATTTTCAGGGCGCCATGAAAGCGGCACTTCACCGT-3′; reverse, 5′-TTGTCGACGGAGCTCTTACGCTTTATTACTCTCACCAT-3′) and inserted into pETM11 vector (EMBL) and expressed in *E.coli* BL21 (DE3) strain. The purification procedures were the same as those of *Vv*Wzb.

### Crystallization and structure determination

Crystallization was conducted with the sitting-drop vapor-diffusion method at 20 °C. Free *Vv*Wzb crystals were grown in drops containing 1.5 μl of protein solution (14 mg/ml in the buffer 10 mM HEPES pH 7.5, 150 mM NaCl, 1 mM DTT) and 1.5 μl of reservoir solution (100 mM HEPES pH7.5, 200 mM NaCl, 31% (w/v) PEG3350). The reservoir solution supplemented with 4% (w/v) PEG3350 was used as cryoprotectant solution. We also crystallized *Vv*Wzb and *Vv*Wzb^C9A^ in the presence of benzylphosphonate. First, benzylphosphonic acid dissolved in 10 mM HEPES pH 7.5, 150 mM NaCl, 1 mM DTT was titrated by NaOH to result in a 300 mM benzylphosphonate solution of pH 7.5. Then, *Vv*Wzb or *Vv*Wzb^C9A^ solution was mixed with the benzylphosphonate solution and incubated at 4 °C for 1 h. Complex crystals were grown in drops containing 1.5 μl of protein solution (14 mg/ml protein in the buffer 10 mM HEPES, 30 mM benzylphophonate, 150 mM NaCl, 1 mM DTT, pH 7.5) and 1.5 μl of reservoir solution (100 mM HEPES pH 7.0, 25% (w/v) PEG3350). The reservoir solution supplemented with 10% glycerol and 30 mM benzylphophonate pH 7.5 was used as cryoprotectant solution. The crystals were shortly soaked in their respective cryoprotectant solutions, prior to flash-freezing in liquid nitrogen for data collection.

The diffraction data were collected at 100 K on the beamlines BL18U1 and BL19U1 of Shanghai Synchrotron Radiation Facility (SSRF) (49, 50). The diffraction data were processed by autoPROC (Global Phasing) calling the programs XDS and Aimless (51, 52, 53). The structure of free *Vv*Wzb was solved by molecular replacement using Phaser (54), with the structure of *Ec*Wzb (PDB ID: 2WMY) as search model. Refinement of the atomic coordinates, B-factors, and TLS parameters using autoBUSTER (55) and model building using Coot (56) were carried out alternately. The chloride ions in free *Vv*Wzb structure were identified on the basis of electron density, anomalous signal, and stereochemistry (57). The ligand-bound structures were solved by molecular replacement using the ligand-free structure as search model. The refinement protocol of the ligand-bound WT *Vv*Wzb is similar to that of the free form. For ligand-bound *Vv*Wzb^C9A^, which diffracted to atomic resolution, the atomic coordinates and anisotropic B-factors were refined using Phenix.refine (58). Ligands were modeled at late stage of refinement, and the geometry restraints of ligands were generated by the GRADE server (http://grade.globalphasing.org). All the crystallographic data are summarized in Table 1.

Structure similarity searching was performed on the Dali webserver (59). Sequence alignment was performed on the ESPript server (60). Protein intereaction interface was analyzed with the PISA program (61). The MolProbity server (62) and other programs in the CCP4 and Phenix packages were also used for structure analysis (63, 64). Pymol (Schrödinger) was used for structure superimposition, surface charge analysis, and structural graphics preparation.

### Phosphatase activity assays

The phosphatase activities of *Vv*Wzb and mutants were measured by an assay using pNPP as substrate. *Vv*Wzb can dephosphorylate pNPP into p-nitrophenol, whose absorbance can be recorded at 405 nm. The determination of kinetic parameters was carried out in a 300 μl reaction system containing 10 mM HEPES pH 7.5, 150 mM NaCl supplemented with 0.45 to 57.6 mM pNPP and different amount of enzyme (100 nM for *Vv*Wzb and its mutants except 400 nM for *Vv*Wzb ^E40-R43del^; 200 nM for *Ec*Wzb and its mutants). Absorbance at 405 nm was recorded continuously on a Cary 60 UV-Vis spectrophotometer (Agilent Technologies) with 10-mm path length cuvettes at 20 °C to obtain the progress curve, which was used to calculate the initial velocity. A standard curve of the product p-nitrophenol was drawn to convert absorbance values to p-nitrophenol concentrations. Kinetics parameters were estimated by nonlinear fitting of the initial velocity *versus* substrate concentration data to the Michaelis–Menten equation (*v*= *V*_*max*_[S]/(*K*_*m*_+[S])) using the Origin program.

The phosphatase activities of *Vv*Wzb and the mutants were also tested using recombinant *Vv*Wzc_453−726_ as substrate. In detail, 10 μM *Vv*Wzc_453−726_ with 3 μM *Vv*Wzb or its mutants was incubated in 10 μl reaction buffer (50 mM HEPES pH 7.5, 150 mM NaCl) at 4 °C for 0.5 h. Then the SDS-PAGE sample buffer was added to terminate the reactions. Samples were heated at 95 °C for 5 min, separated on 12% SDS-PAGE gels, and transferred onto PVDF membranes using a wet transferring protocol with constant current 200 mA. Membranes were firstly incubated with monoclonal anti-phosphotyrosine antibody (1:2000 diluted ab10321; Abcam), followed by incubation with goat anti-mouse IgG-HRP (1:5000 diluted CW0102S; CWBIO). Immunodetection was performed using the DAB kit (CW0125S; CWBIO). The intensity of the bands was quantified using the Image J software. The activity was evaluated by the intensity difference between samples and the control.

### Phylogenetic analysis

The rootless protein phylogenetic tree was constructed by MEGA6 software using the neighbor-joining method. In total, 26 representative LMWPTP sequences including eukaryotic and prokaryotic ones were used for analysis (with NCBI numbers in the parentheses): BPTP *Bos taurus* (NP_776403.1), HCPTPA human (NP_004291.1), Stp1 *S. pombe* (NP_001342920.1), LMWPTP *Mus* (NP_001103709.1), LMWPTP1 *V. cholerae* (WP_001889901.1), PTP *H. felis* (OOS02738.1), MptpA *Mycobacterium tuberculosis* (WP_003411510.1), LMWPTP *S. humiferus* (WP_003975011.1), LMWPTP *A. lwoffii* (WP_004278810.1), PTP *Erwinia amylovora* (WP_004158327.1), LMWPTP *R. solanacearum* (WP_193029717.1), Wzb *S. sonnei* (EFW8105757.1), Wzb *E. coli* (NP_416565.1), Wzb *S. enterica* (WP_000482223.1), Wzb *K. pneumoniae* (AAD30006.1), Wzb *Escherichia hormaechei* (ESL87399.1), PTP *C. sakazakii* (KDP98850.1), Wzb *V. vulnificus* (WP_017420711.1), LMWPTP *V. cholerae* (WP_114808776.1), Wzb *Vibrio parahaemolyticus* (WP_005458338.1), Wzb *Vibrio harveyi* (WP_021018098.1), Wzb *Vibrio crassostreae* (WP_055320062.1), PTP *A. sifiae* (WP_061013219.1), PTP *P. damselae* (WP_065171812.1), LMWPTP *E. coli* (WP_122056837.1), and LMWPTP *G. parasuis* (WP_010785901.1). One thousand bootstrap replicates were used to test the inferred phylogeny relationship. The final clustering result was generated and displayed with radiation.

## Data availability

The coordinates and diffraction data of the free *Vv*Wzb, *Vv*Wzb-benzylphosphonate, and *Vv*Wzb^C9A^-phosphate have been deposited in the PDB (www.rcsb.org) with accession numbers of 7DHD, 7DHE, and 7DHF, respectively. All remaining data are contained within the article and the supporting information file.

## Conflict of interest

The authors declare that they have no conflicts of interest with the contents of this article.
