# Supplementary material for: Wzb of Vibrio vulnificus represents a new group of low-molecular-weight protein tyrosine phosphatases with a unique insertion in the W-loop
Source: J Biol Chem. 2021 Jan 12;296:100280. doi: 10.1016/j.jbc.2021.100280 (PMC7948962; doi:10.1016/j.jbc.2021.100280)
Supplement: Supplemental Figures S1–S3 [file mmc1.docx]

**Wzb of *Vibrio vulnificus* represents a new group of low molecular weight protein tyrosine phosphatases with a unique insertion in the W-loop**

Xin Wang^a, b, c,^ , Qingjun Ma^a, b, c, d^ *

^a^ Key Laboratory of Experimental Marine Biology, Institute of Oceanology, Chinese Academy of Sciences, Qingdao, China.

^b^ Laboratory for Marine Biology and Biotechnology, Pilot National Laboratory for Marine Science and Technology (Qingdao), China.

^c^ University of Chinese Academy of Sciences, Beijing, China.

^d^ Center for Ocean Mega-Science, Chinese Academy of Sciences, Qingdao, China.

* Corresponding author: Qingjun Ma

E-mail: qma@qdio.ac.cn

**Running title:** *V. vulnificus* Wzb structures

This file contains supporting information: figures S1–S3.


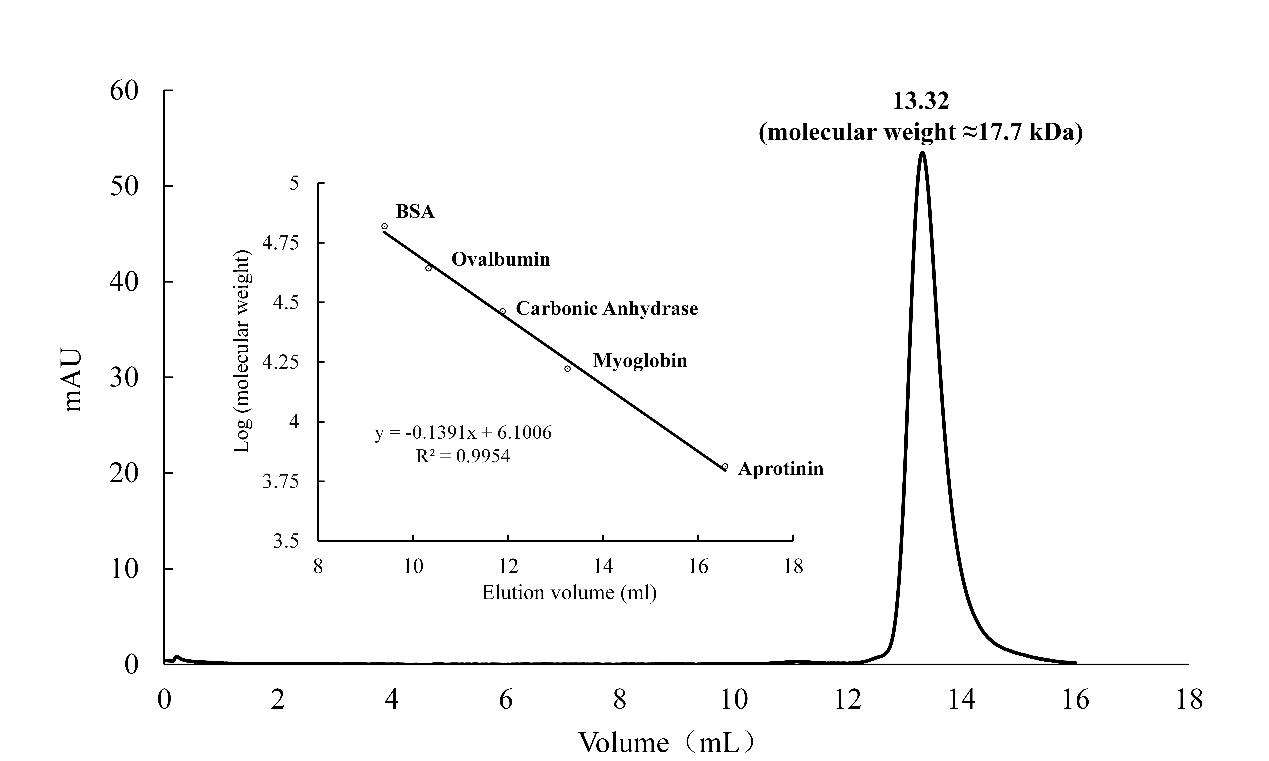
**Figure S1.** **Gel filtration profile of *Vv*Wzb.** The apparent molecular weight of *Vv*Wzb on a Superdex^TM^ 75 Increase 10/300 GL (GE Healthcare) is estimated to be 17.7 kDa, corresponding to a monomeric form (theoretical molecular weight: 16.1 kDa), The inner panel is the standard curve calculated from the elution volumes of standard protein samples (BSA 66 kDa; Ovalbumin 44 kDa; Carbonic Anhydrase 29 kDa; Myohemoglobin 16.7 kDa and Aprotinin 6.5 kDa).


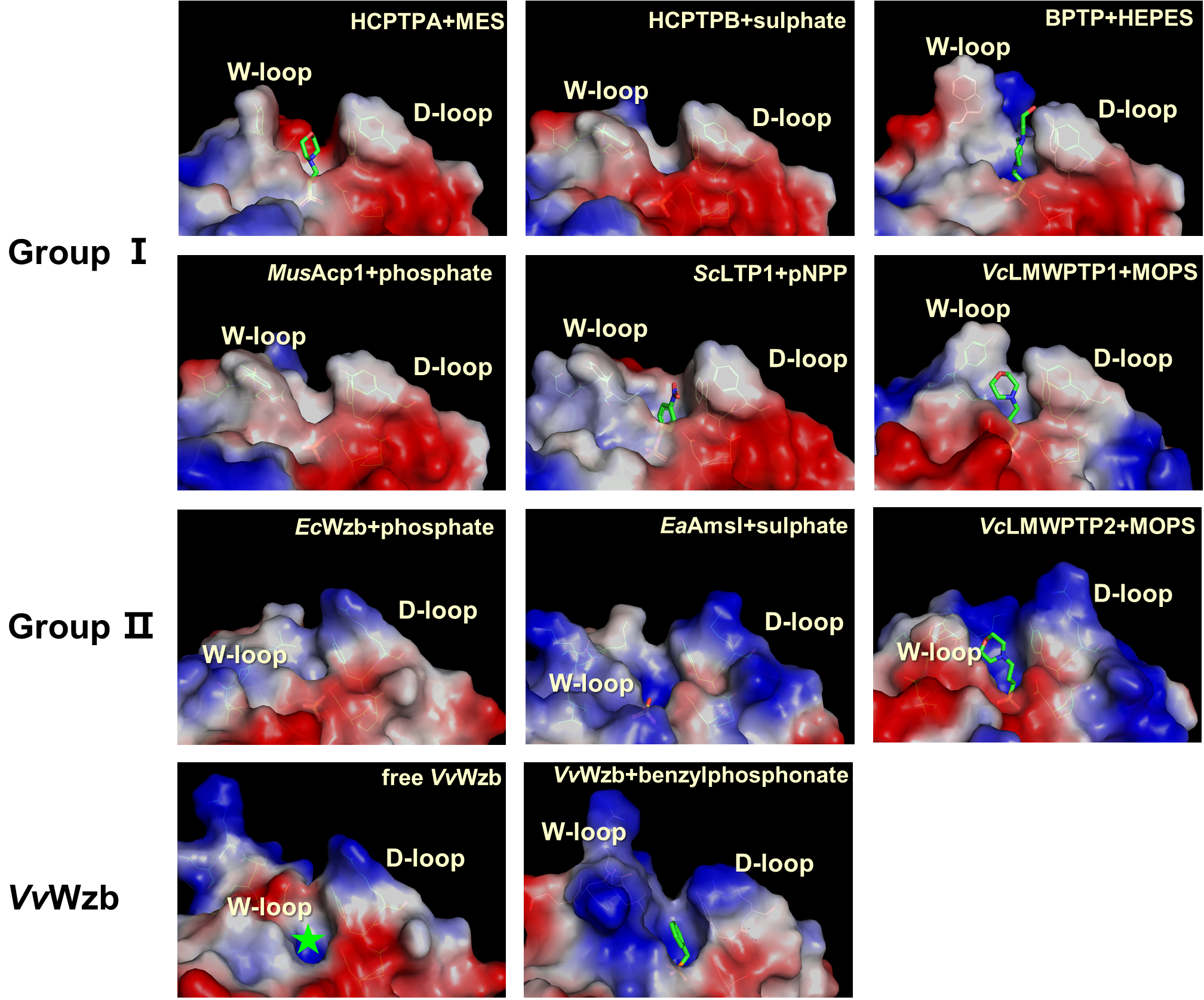


**Figure S2. Shape and surface electrostatic potential of the active site crevices of different LMWPTPs.** Group I LMWPTPs include HCPTPA with MES (PDB ID: 5PNT), HCPTPB with sulphate (PDB ID: 1XWW), BPTP with HEPES (PDB ID: 1DG9), *Mus*Acp1 with phosphate (PDB ID: 2P4U), *Sc*LTP1 with pNPP (PDB ID: 1D1Q), and *Vc*LMWPTP1 in complex with MOPS (PDB ID: 4LRQ); Group II LMWPTPs include *Ec*Wzb in complex with phosphate (PDB ID: 2WJA), *Ea*AmsI with sulphate (PDB ID: 4D74), and *Vc*LMWPTP2 with MOPS (PDB ID: 5Z3M). Free *Vv*Wzb and its complex with benzylphosphonate are shown at the bottom. The active site position in free *Vv*Wzb is indicated by a green star. The negatively, neutrally and positively charged surfaces are shown in red, white and blue, respectively. The surfaces are set with 20% transparency to display the residues constituting the D- and W-loop walls.


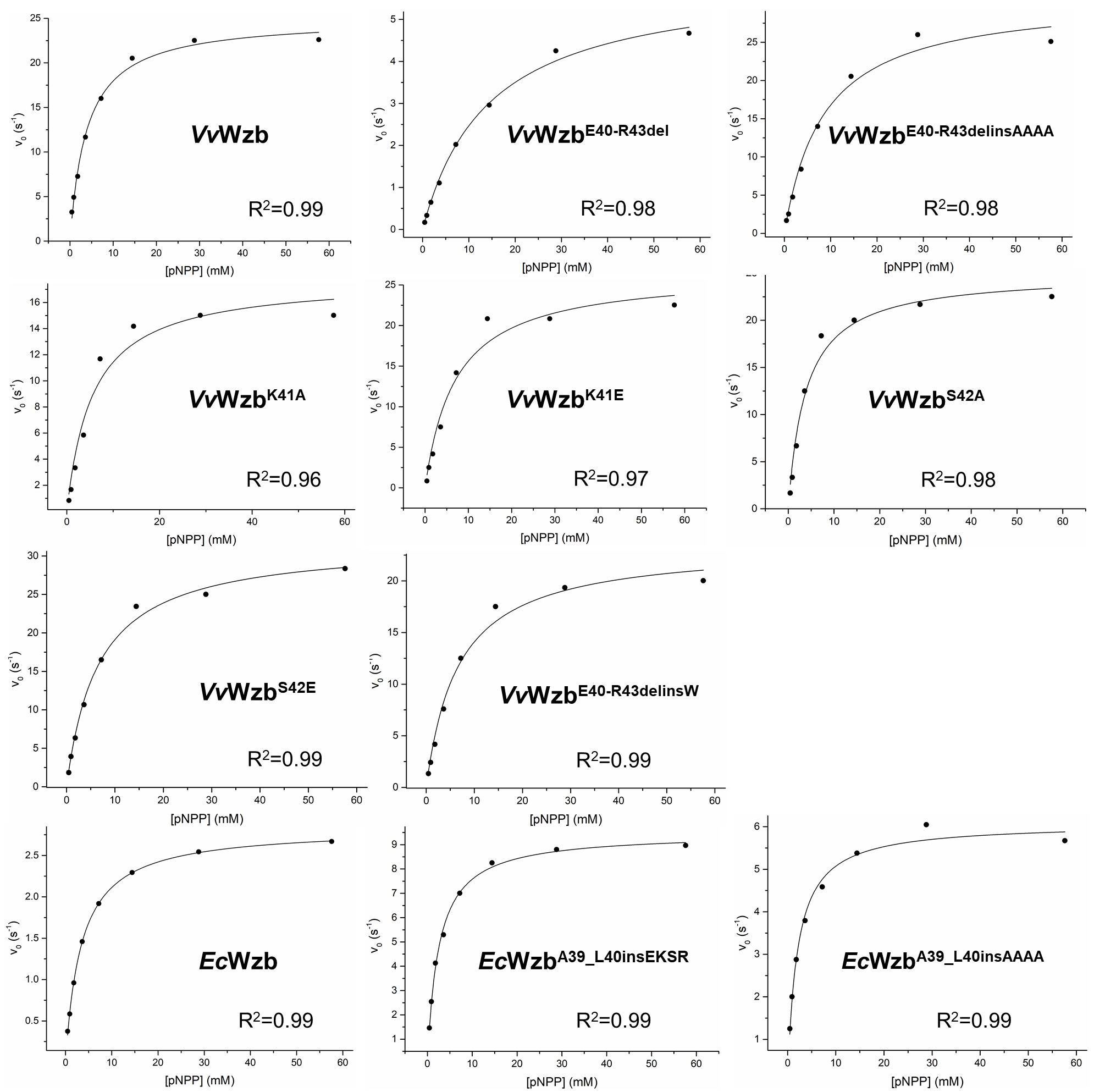


**Figure S3. Representative initial velocity versus substrate concentration plots for the phosphatase assay using pNPP as substrate.** The kinetics parameters estimated from the nonlinear fitting of the data to the Michaelis-Menten equation are listed in Table 2.
